# Molecular Remodeling of Milk Fat Globules Induced by Centrifugation: Insights from Deep Learning-Based Detection of Milk Adulteration

**DOI:** 10.3390/ijms262411919

**Published:** 2025-12-10

**Authors:** Grzegorz Gwardys, Grzegorz Grodkowski, Piotr Kostusiak, Wojciech Mendelowski, Jan Slósarz, Michał Satława, Bartłomiej Śmietanka, Krzysztof Gwardys, Marcin Gołębiewski, Kamila Puppel

**Affiliations:** 1 Promity sp. z o.o., ul. Wiejska 14/25, 00-490 Warsaw, Poland; grzegorz.gwardys@pw.edu.pl (G.G.); wojciech.mendelowski@promity.com (W.M.); michal.satlawa@promity.com (M.S.); bartlomiej.smietanka@promity.pl (B.Ś.); krzysztof.gwardys@promity.com (K.G.); 2 Institute of Animal Science, Warsaw University of Life Sciences, Ciszewskiego 8, 02-786 Warsaw, Poland; grzegorz_grodkowski@sggw.edu.pl (G.G.); piotr_kostusiak@sggw.edu.pl (P.K.); jan_slosarz@sggw.edu.pl (J.S.); marcin_golebiewski@sggw.edu.pl (M.G.)

**Keywords:** artificial intelligence, machine learning, milk, centrifugation, casein micelles, somatic cell count, deep learning, microscopy, food adulteration

## Abstract

Milk adulteration through centrifugation, which artificially reduces the somatic cell count (SCC), represents a significant challenge to food authenticity and public health. This fraudulent practice alters the native molecular architecture of milk, masking inflammatory conditions such as subclinical mastitis and distorting product quality. Conventional analytical and microscopic techniques remain insufficiently sensitive to detect the subtle physicochemical changes associated with centrifugation, highlighting the need for molecular-level, data-driven diagnostics. The dataset included 128 paired raw milk samples and approximately 25,000 bright-field micrographs acquired across multiple microscopes, of which 95% were confirmed to be of high quality. In this study, advanced machine learning (ML) and deep learning (DL) approaches were applied to identify centrifugation-induced alterations in raw milk microstructure. Bright-field micrographs (pixel size 0.27 µm) of paired unprocessed and centrifuged samples were obtained under standardized optical conditions and analyzed using convolutional neural networks (ResNet-18/50, Inception-v3, Xception, NasNet-Mobile) and hybrid attention architectures (MaxViT, CoAtNet). Model performance was evaluated using the harmonic average of recalls across five micrographs per sample (HAR_5_). Human microscopy experts (n = 4) achieved only 18% classification accuracy—below the random baseline (25%)—confirming that centrifugation-induced modifications are not visually discernible. In contrast, DL architectures reached up to 97% accuracy (HAR_5_, Xception), successfully identifying subtle molecular cues. Class activation and sensitivity analyses indicated that models focused not on milk fat globule (MFG) boundaries but on high-frequency nanoscale variations related to the reorganization of casein micelles and solid non-fat fractions. The findings strongly suggest that centrifugation adulteration constitutes a molecular reorganization event rather than a morphological alteration. The integration of optical microscopy with AI-driven molecular analytics establishes deep learning as a precise and objective tool for detecting fraudulent milk processing and improving food integrity diagnostics.

## 1. Introduction

Milk is a complex colloidal system whose functional properties depend on the interplay between lipids, proteins, carbohydrates, and cellular components. Because this molecular architecture is highly sensitive to physicochemical disturbances, even subtle manipulations can alter its native organization. Centrifugation used as an adulteration method is particularly problematic: by artificially reducing somatic cell count (SCC), it masks subclinical mastitis and produces milk that appears normal macroscopically yet differs at the molecular level [1]. Somatic cells supply enzymes, cytokines, and antioxidant molecules that contribute to proteolytic and oxidative balance; their removal disrupts this equilibrium and generates milk that no longer reflects its true physiological state.

At the subcellular level, centrifugation exerts combined shear and gravitational stress that disrupts the milk fat globule (MFG) and its surrounding milk fat globule membrane (MFGM). The MFGM constitutes a trilaminar interface composed of phospholipids, glycoproteins, and integral membrane proteins—such as butyrophilin, xanthine oxidase, and adipophilin—that ensure the structural and oxidative stability of the emulsion [2]. Previous studies have shown that centrifugal forces can promote partial delamination of phospholipids, denaturation of interfacial proteins, and redistribution of amphiphilic molecules within the MFG membrane [2,3]. Such molecular rearrangements have been reported to coincide with changes in lipid oxidation, surface charge, and the integrity of the surrounding casein micellar matrix [3]. In the present study, these mechanisms are referenced as literature-based background rather than directly measured effects. These processes have been proposed to influence the supramolecular architecture and rheological behavior of milk, although they are not directly assessed in our optical dataset. The concomitant removal of somatic and bacterial cells reduces enzymatic and proteolytic activity, further destabilizing the post-secretory equilibrium of the milk matrix. These cumulative effects manifest as subtle changes in supramolecular architecture and rheological properties that are typically imperceptible by human observation or conventional analytical assays. Classical cytometric and spectroscopic methods lack the sensitivity to resolve nanoscale modifications occurring at the MFG-MFGM interface. In this context, machine learning (ML) and artificial intelligence (AI) offer a transformative analytical paradigm capable of recognizing multidimensional molecular patterns within complex biological images. By integrating image-derived biophysical descriptors—such as texture anisotropy, fractal geometry, and microstructural variance—ML systems can distinguish unprocessed from centrifuged milk with accuracies exceeding 90%, acting as computational molecular sensors that infer nanoscale organization from optical data.

From a biophysical perspective, milk represents a metastable colloidal suspension of triacylglycerol droplets, casein micelles, and whey proteins maintained in dynamic equilibrium through reversible molecular interactions [4,5]. Mechanical perturbation by centrifugation disrupts this equilibrium, inducing heterogeneous redistribution of MFG populations and modifying the surface composition and curvature elasticity of the MFGM. Such perturbations alter zeta potential, interfacial packing density, and lipid phase transition dynamics—parameters directly linked to milk’s colloidal stability. Consequently, the identification of centrifugation-induced molecular markers requires analytical methodologies that integrate physical optics, structural biochemistry, and data-driven pattern recognition.

Despite strict legislative frameworks, including Regulation (EC) No. 853/2004—which defines raw milk as a product free from mechanical modification [6]—there remains a lack of molecular-scale diagnostic tools capable of reliably detecting centrifugation. The dual nature of centrifugation—as both a legitimate technological process and a potential vector for fraud—further complicates regulatory enforcement. Establishing molecularly grounded diagnostic indicators of centrifugation is therefore essential for ensuring product integrity, verifying authenticity, and protecting consumers from deceptive processing practices.

Although the molecular effects of mechanical processing have been partly described in biochemical and colloidal studies, no previous work has examined whether centrifugation leaves a detectable optical fingerprint that can be identified computationally. This represents an important gap, as the dairy sector lacks rapid, objective, and non-invasive tools for identifying mechanically modified raw milk. Developing such methods is essential for ensuring authenticity and regulatory compliance across both farm-level and industrial settings. To address this gap, the present study integrates high-resolution microscopy, quantitative image analysis, and deep learning–based modeling to characterize the molecular consequences of centrifugation. Specifically, we investigate whether centrifugation perturbs the supramolecular organization of milk fat globules, alters lipid–protein interactions within the milk fat globule membrane, and generates submicron-scale heterogeneity detectable through computational analysis. We formulated two working hypotheses: (H1) centrifugation does not induce visually discernible morphological changes in milk fat globules, and (H2) centrifugation produces molecular-level reorganizations—particularly within casein micelles and solid non-fat components—that manifest as subtle optical signatures identifiable by deep learning. To our knowledge, this is the first study to evaluate centrifugation-induced molecular reorganization directly from bright-field micrographs using artificial intelligence. By testing hypotheses H1 and H2, we aim to determine whether AI-based analysis can reveal subvisual structural cues that escape human detection, thereby establishing a foundation for objective diagnostics of centrifugation-related adulteration.

## 2. Results

### 2.1. Experts Trial

A controlled trial involving human microscopy experts was conducted to evaluate whether their classification performance differed significantly from that of the neural models. The experimental setup was designed to mirror the data and conditions provided to the models as closely as possible. Images were standardized to a pixel resolution of 0.27 µm, and each participant received five randomly selected micrographs (608 × 608 px crops) per sample. The test dataset comprised 128 samples in total, with each of the four experts evaluating 32 unique samples (Table 1).

The results indicate that expert predictions exhibited a bias toward centrifuged milk samples (72 vs. 56 predictions) and toward healthy samples (86 vs. 42 predictions). The overall human accuracy rate (HAR) was 18%, which is lower than the 25% expected from a random uniform classifier. When analyzed by individual condition, HAR values for centrifugation and mastitis classification were 52% and 39%, respectively, compared to 50% for random uniform performance.

These results suggest that even trained human observers encountered difficulty distinguishing between centrifuged and unprocessed milk micrographs, particularly when inflammatory or compositional changes overlapped. This outcome emphasizes the necessity of advanced computational models capable of identifying subtle molecular or structural cues that are not easily perceptible through direct human inspection.

### 2.2. Modeling

When trained to detect a single feature, the attentive neural architectures exhibited bistable behavior, converging toward models that consistently classified all samples as either centrifuged or unprocessed. This pattern suggested instability in the internal attention dynamics under constrained target dimensionality. The situation changed markedly when the models were trained using multi-class power-set targets, which expanded the label space to include combinations of attributes. Under this regime, both MaxViT and CoAtNet architectures achieved stable convergence with only occasional oscillations in class attribution, although their overall performance remained below the mean accuracy of convolutional baselines. Larger attention-based architectures could not be successfully trained due to out-of-memory (OOM) limitations, indicating that their potential performance may improve with access to higher-capacity computational resources.

Using power-set encoding for multi-class training functioned as a regularization mechanism for attentive networks. However, contrary to expectations, it did not uniformly enhance all individual feature detections. Instead, the resulting accuracies resembled a harmonic mean of the corresponding one-dimensional classifiers. For example, because no model reliably detected mastitis, joint training on the power-set {centrifugation, mastitis} decreased the centrifugation detection score (HAR_5_) to approximately 60%, comparable to the performance for mastitis detection alone. Conversely, when the models were trained on the pair {centrifugation, freezing}, where freezing alone could be recognized with a HAR_5_ of ~98%, the combined training elevated the centrifugation score to around 90%, but simultaneously reduced the freezing accuracy to a similar level. This reciprocal trade-off suggests that feature entanglement within the shared representation layer constrained independent classification performance.

Randomness in data partitioning accounted for the majority of the observed variance in the averaged HAR values across sessions, whereas stochasticity within training processes (sample ordering, cropping, or augmentation) contributed only approximately 1%. Consequently, extreme results reported in Table 2, especially those deviating beyond one standard deviation from the session mean, likely resulted from fortuitous data splits rather than superior model generalization. Specifically, high validation and test scores may have occurred when difficult cases were disproportionately allocated to the training set, thereby simplifying evaluation. Although one could argue that exposure to more challenging samples improves model robustness, such distributions risk overfitting, where the network effectively memorizes complex patterns in the training data without developing true generalization capacity.

Table 2 summarizes the comparative performance of the evaluated architectures in the simultaneous detection of centrifugation and freezing. Results are expressed as HAR_5_ values obtained from cross-validation sessions involving six independently trained models per architecture. The reported uncertainties represent standard deviations within each session.

### 2.3. Class Activation and Sensitivity Maps

**Figure 1:**
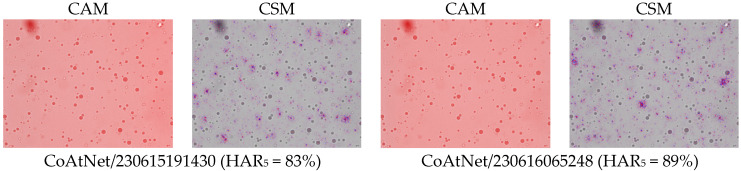

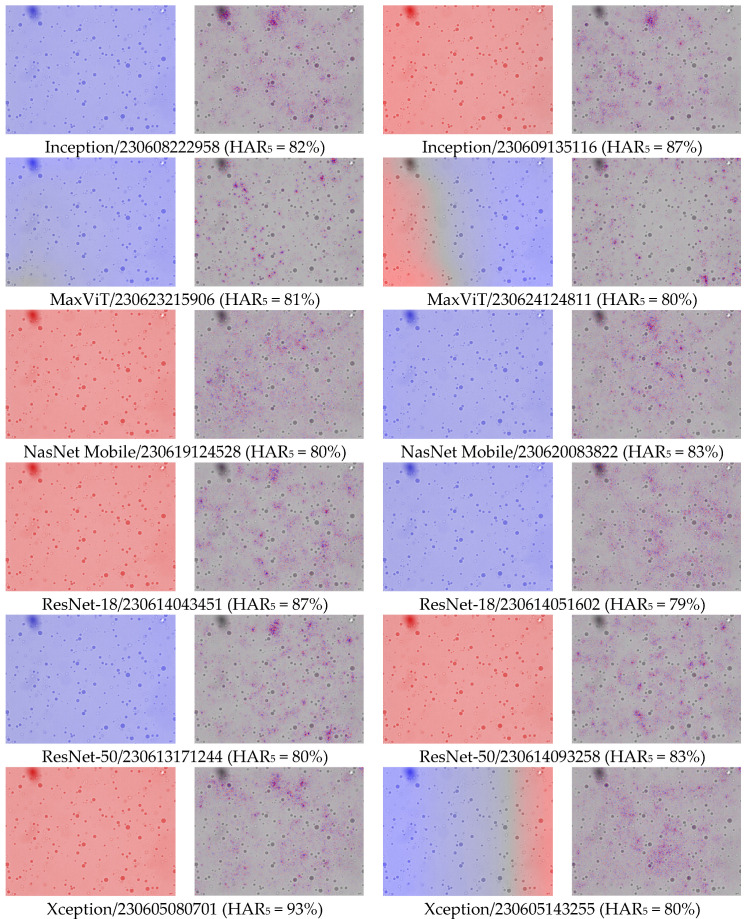
Examples of the differences in CAM and CSM (gradient of *y*) between models and architectures. Percentage numbers in brackets are HAR_5_ measures on the test set. To make the comparison meaningful, model inputs were identical in all cases. The category-colored CAM and CSM are overlain on the input image. The derivative oscillates between neighboring pixels, hence, the red (positive) and blue (negative) values often look purple from afar. Notice that most of the CSM coloring is around points that are hardly distinguishable from the background (all micrographs were acquired at 40× optical magnification (pixel size: 0.27 µm). Since all sub-figures were obtained under identical imaging conditions, the same magnification/scale applies to all panels).

The first and third columns of Figure 1 give examples of class activation and sensitivity maps (CAM and CSM). The second and fourth columns display examples of the gradient $\nabla_{x}y$ for different architectures and trained models. First of all, we observe that all gradients vanish at the MFG boundaries. This is somewhat surprising because, according to our initial hypothesis (H2), the membranes should be damaged due to centrifugation. This observation also holds true when using alternative CSM methods.

Second, the gradient varies so frequently between adjacent points that the red and blue coloring appears violet from a distance. To a large extent, this is the effect of the non-differentiable ReLU activations and MaxPool functions used throughout the studied architectures. But not only this: despite the gray-scale input, all models were in fact training three color channels that were fed identical intensities; and it turns out that each became a different sub-model, with evidently different CSM. Averaging over these RGB channels, as performed in Figure 1, makes the variability even more frequent. Furthermore, the average is several orders of magnitude smaller than the second moment, thus the gradient signal is almost centered, $E^{2}(\nabla_{x}y)\approx E({\left(\nabla_{x}y\right)}^{2})$. This would make one rightfully question the meaning of the gradient sign, as the signal-to-noise ratio seems very low indeed.

**Figure 2:**
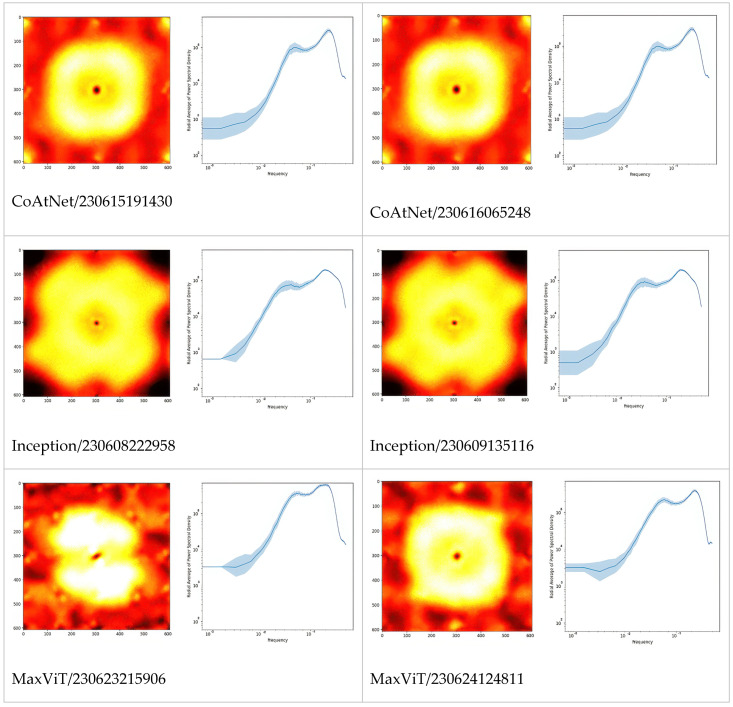

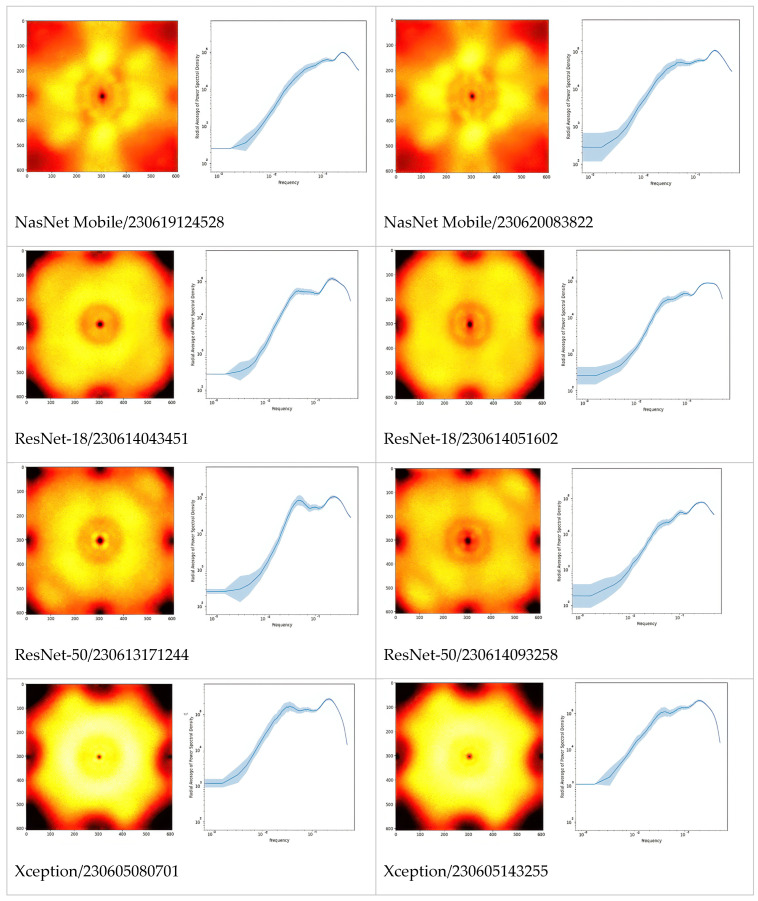
Fourier power spectral densities (PSD, 1st and 3rd columns), and their radial averages (RAPSD, 2nd and 4th columns) of the gradient CSM. For each architecture, the two top models are presented to illustrate the level of difference between training sessions. The PSD were obtained by first normalizing to total power, then averaging over a fixed set of samples and microscopes. The shaded areas in the graphs are standard deviations of logarithmic RAPSD. Since the frequency is inversely proportional to the scale, the highest frequencies correspond to smallest features, and vice versa.

In Figure 2, we extended the comparison between models further by picturing PSD of Fourier-transformed CSM. Apparently, these are the fingerprints of the models, especially architectures which can be differentiated just by looking at the PSD or even their radially averaged PSD (RAPSD). Similarities were evident between ResNet-18 and ResNet-50, and also between Inception-v3 and its successor, Xception. All of the RAPSD had peaks around a similar frequency lying before the image resolution’s maximum, meaning that the most important features were ~1.6 µm across, while the second (from the right, and often from the top) peak corresponded to about ~9 µm.

### 2.4. Classification Based on MFG Distributions 

**Figure 3:**
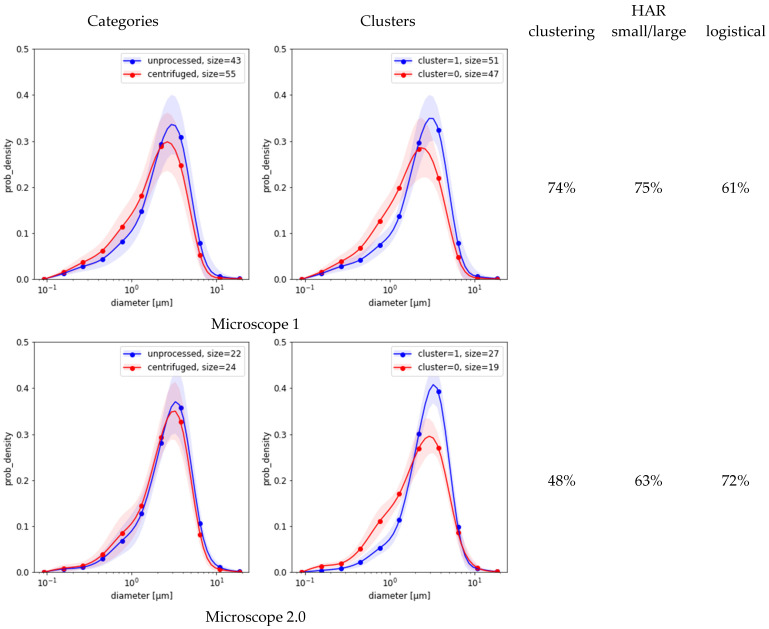

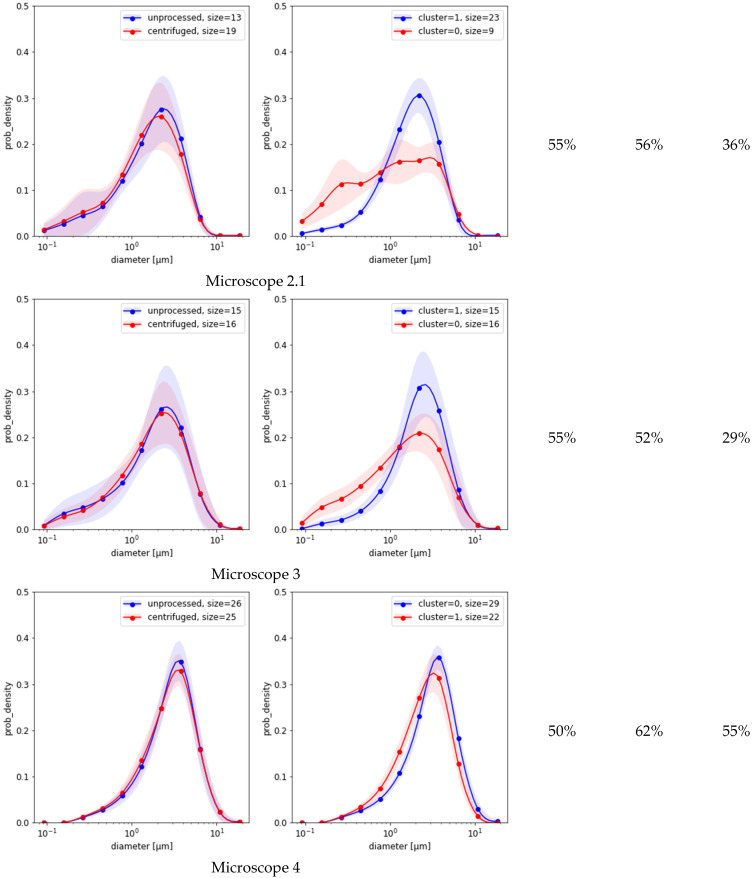
MFG distributions split into categories (**left charts**) vs. clustering of the same distribution dataset (**right charts**). Shaded areas are standard deviation margins. The columns to the right of the charts provide HAR measurements of clustering as a detection means (left) and an estimated classification score based on small/large ratio (middle, Equation (4)).

Figure 3 compares the results of the three methods based on MFG segmentation data. First, the spectral clustering of the MFGD demonstrates that for each microscope there were two major groups of distributions, each with reduced variance (Figure 3, second column). Separation worked best for microscope 1, achieving a HAR measure of 74% when compared against ground truth; but in the remaining four cases it was indistinguishable from random (Figure 3, third column). Evidently, the detected differences between distribution shapes are associated with features unrelated to centrifugation. Second, simple separation based on the number of small-vs.-large MFG gave better results than clustering (Figure 3, fourth column). Third, the simple logistic classification of MFG data obtained from sample micrograph segmentation showed that circularity or eccentricity were uncorrelated with centrifugation. Depending on which microscope was used for the test, the HAR_5_ detection scores based on MFG diameter distributions alone varied between 29%÷73% as listed in the last column of Figure 3. None of these methods could explain the much higher scores achieved by neural models.

**Figure 4:**
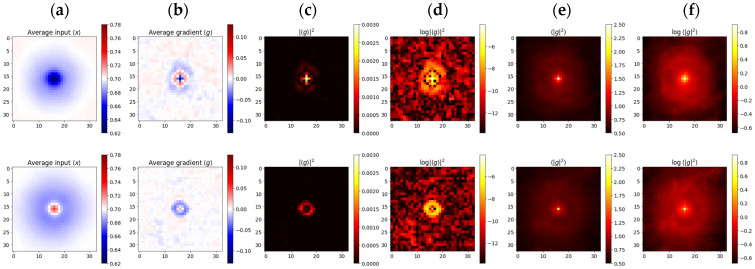
Weighted averages of input points and gradients *g* separated into dark (top) and bright (bottom) rows. (**a**) coherent average of the input <*x*>; (**b**–**d**) coherent average of the gradient <*g*> and its power |<*g*>|^2^; (**e**,**f**) incoherent averages of <|*g*|^2^>.

Figure 4 presents the results of selective input x and gradient $g\equiv \nabla_{x}y$ averaging, aimed at detecting the sensitivity of models to tiny pixel-sized features. Dark dots were pit-shaped, while bright ones were hat-shaped (Figure 4a). Many of the bright objects turned out to be dark objects, just slightly out of focus; therefore, models developed similar associations. Nevertheless, the average gradients of bright objects were evidently weaker, meaning that the sensitivity was almost balanced, while in the case of dark objects, there was a bias towards the negative (Figure 4b). Models learned to develop oscillating response functions similar to those found in ganglion cells, which, in turn, likely follow optics’ point-spread functions (PSF) (Figure 4b–d). These Airy-like patterns were almost invisible in the (incoherent) averages $\langle {\left|g\right|}^{2}\rangle ,$ suggesting that the gradient sign, despite being deterministic, could well be treated as a random event, of a certain distribution, over the input image (Figure 4e,f). Probabilities close to {1,½,0} led to averages of $\left\{1,0,-1\right\},$ respectively, producing the interference rings visible in columns 6b-d. Would the sign be a definite function, then there would also have to be a transition boundary between +1 and −1, and we would clearly observe corresponding dark rings in columns Figure 4e,f.

## 3. Discussion

Milk represents a paradigmatic example of a biologically engineered colloidal system, in which structural integrity and metabolic function emerge from the hierarchical assembly of lipids, proteins, and cells into a finely tuned molecular network. The present findings demonstrate that centrifugation—a process deceptively simple in physical principle—elicits complex molecular perturbations extending far beyond the visible microstructure. When applied fraudulently, centrifugation reconfigures the molecular architecture of milk, selectively redistributing its colloidal components and disrupting proteolipid equilibrium. These molecular fingerprints, imperceptible to human vision, can be detected and decoded by deep learning (DL) frameworks functioning as artificial molecular sensors. The evidence thus establishes that milk adulteration is a molecular event rather than a mere compositional irregularity.

The results of the Experts Trial directly refute the assumption that centrifugation-induced alterations are morphologically perceptible. The harmonic average recall (HAR) of only 18%-well below random baseline-shows that even trained microscopists could not differentiate adulterated from genuine samples under standardized conditions. This suggests that the inability of human experts to discern structural differences reflects the subvisual nature of centrifugation-induced changes. This finding invalidates Hypothesis 1 (H1) and demonstrates that centrifugation does not produce visible morphological deformation of milk fat globules (MFGs). Instead, it induces molecular-scale reorganizations that occur below the threshold of visual discrimination. This observation is consistent with earlier reports by Deeth and Datta [7] and Shi et al. [8], who noted that centrifugal acceleration removes dense micellar and cellular phases while leaving lipid droplets macroscopically intact. Consequently, the processed milk remains optically deceptive despite a disrupted internal composition.

In contrast, deep neural architectures achieved high classification accuracies (HAR_5_ up to 97% in Xception and 90% in ResNet-18), confirming that machine learning models extract discriminative molecular information inaccessible to human observers. Class activation and sensitivity maps (Figure 3) indicate that the models do not focus on MFG membranes-the expected sites of deformation-but rather on diffuse submicron optical fluctuations dispersed throughout the image. Such redistribution patterns imply that centrifugation modifies the local optical coherence of the milk matrix. These findings invalidate Hypothesis 2 (H2) and suggest that discriminative features arise not from lipid boundaries, but from the dynamic reorganization of casein micelles and solid non-fat (SNF) fractions within the colloidal matrix. This interpretation aligns with the molecular mechanisms described by Vanderghem et al. [2] and Bourlieu and Michalski [3], who showed that mechanical stress delaminates the phospholipid trilayer of the MFG membrane, denatures amphiphilic proteins, and perturbs lipid-protein associations without producing macroscopic collapse. The DL models appear to have internalized these optical-molecular couplings, effectively identifying refractive irregularities caused by nanoscale phase redistribution.

Fourier-transform analyses of the gradient fields (Figure 4) provide quantitative evidence for these phenomena. The networks respond to spatial frequencies of approximately 1.6 µm and 9 µm-values that correspond not to the mean MFG diameter (0.1–15 µm) [3,4] but to the extrema of the MFG size distribution’s derivative. Such characteristic scales are consistent with the reported bimodal distribution of milk fat globules and the clustering behavior of casein aggregates [3,9]. The persistence of these spectral peaks across architectures suggests that the models capture differences in the rate of change in MFG sizes rather than their absolute dimensions. This indicates that the observed frequencies likely reflect gradients in MFG size transitions-zones where small and large globules coexist and create sharp optical discontinuities-rather than individual droplet diameters themselves. Such redistribution patterns imply that centrifugation modifies the spatial coherence and optical phase continuity of the milk matrix. Therefore, centrifugation-induced adulteration can be conceptualized as a nanoscale remodeling process, detectable through its distinct optical and spectral signatures.

This molecular interpretation is consistent with the physical principles governing micellar behavior. Casein micelles, with diameters of 0.05–0.25 µm [9], are denser and more cohesive than lipid droplets. Under centrifugal force, larger micelles sediment preferentially, leading to selective depletion of high-mass aggregates and enrichment of smaller micelles. This process alters light-scattering properties, producing the high-frequency optical noise that neural models utilize for classification. The Airy-like diffraction patterns observed in class sensitivity maps (Figure 4) further indicate that these networks operate near the diffraction limit, effectively reconstructing the microscope’s point-spread function (PSF). Such behavior suggests that the discriminative information originates from features smaller than the imaging resolution, providing direct evidence that centrifugation modifies milk at the molecular and submicron scale. Thus, neural networks function not merely as pattern recognizers but as computational microscopes capable of translating imperceptible nanoscale variance into quantifiable molecular information.

From a biochemical perspective, centrifugation perturbs the metastable colloidal equilibrium that underpins milk’s molecular organization. The balance among triacylglycerols, phospholipids, and proteins depends on electrostatic, hydrogen-bonding, and hydrophobic interactions [3,5]. Mechanical acceleration disrupts these non-covalent networks, while the removal of somatic and bacterial cells depletes enzymes and cytokines essential for proteomic and oxidative homeostasis [1]. As a result, the milk matrix undergoes coupled physicochemical and biochemical rearrangements, manifesting as nanoscale fluctuations in refractive index and density. These optical interference patterns constitute a molecular fingerprint of centrifugation, detectable by deep learning but invisible to conventional microscopy.

A particularly relevant comparison arises between centrifugation and mastitis. Mastitic milk exhibits micellar fragmentation, enhanced proteolysis, and a shift toward smaller micelles [10,11]. Centrifugation amplifies these effects, driving the colloidal system toward reduced heterogeneity and increased micellar uniformity. This convergence of mechanical and pathological alterations explains the reduced false-negative rate observed in ML detection: both processes disrupt casein-lipid equilibrium and produce similar high-frequency optical signatures. These findings reinforce a fundamental molecular principle-perturbations that destabilize casein-lipid-aqueous interfaces consistently manifest as reproducible optical and spectral fingerprints.

Conversely, MFG-based parameters proved unreliable as molecular discriminants owing to their sensitivity to external variation. The MFG size distribution (MFGD) is modulated by breed, diet, lactation phase, and environmental conditions [12,13,14,15]. Dietary supplementation with conjugated linoleic acid (CLA) substantially alters both MFG abundance and size distribution [16], making them unstable indicators of adulteration. The low saliency of lipid-rich regions in DL activation maps supports this conclusion: the models assign minimal diagnostic weight to the lipid phase, focusing instead on proteinaceous and colloidal domains. The decisive molecular signature of centrifugation therefore resides in micellar reorganization rather than gross lipid morphology.

Collectively, these findings establish that centrifugation-induced milk adulteration constitutes a molecular restructuring event that redefines the colloidal equilibrium and optical coherence of the milk matrix. Deep learning architectures, through their ability to internalize high-dimensional correlations between optical variance and molecular organization, transcend the perceptual limitations of human microscopy. They operate as data-driven molecular analyzers, capable of inferring nanoscale reorganization directly from imaging data. This integration of optical physics, molecular biochemistry, and artificial intelligence delineates a new frontier in computational molecular diagnostics, offering a robust framework for real-time detection of food adulteration, authentication of biological materials, and elucidation of structure-function relationships in complex biomolecular systems.

Although certain factors are often considered potential limitations in imaging-based studies, several measures implemented in the present work effectively minimized their influence. Variability across microscope–camera systems was addressed through rigorous color and geometric normalization, combined with advanced augmentation procedures that simulated realistic optical deviations. These steps substantially reduced device-dependent bias and ensured stable performance across different imaging conditions. Similarly, any potential impact of Sudan III staining was controlled by using a standardized protocol with consistent concentrations and exposure times, while the deep learning models primarily relied on textural and spectral features that are robust to minor intensity variations. Although complementary physicochemical techniques such as TEM or DLS could further enrich the mechanistic interpretation, the multi-microscope validation performed here demonstrated that the optical–computational signatures identified by the models were highly reproducible. Together, these methodological safeguards substantially mitigate factors that might otherwise be perceived as limitations, supporting the reliability and robustness of the presented findings.

## 4. Materials and Methods

### 4.1. Samples, Storage, Centrifugation, and Images

All milk samples were sourced exclusively from local dairy farms cooperating with the Institute of Animal Science, Warsaw University of Life Sciences (SGGW). The material consisted of fresh, raw farm milk collected directly after milking into sterile containers, transported under cooled conditions, and processed immediately upon arrival or stored at 4 °C for no longer than 1–2 days when immediate processing was not possible. Some samples were frozen during the early stage of data acquisition and were subsequently imaged; however, these frozen samples were not used in the centrifugation-detection analyses. As described in Section 4.4, they were removed during data filtering prior to model training. Importantly, all global normalization parameters (channel-wise means and standard deviations) were calculated solely from non-frozen samples, ensuring that frozen material did not influence the preprocessing or statistical characteristics of the dataset used for centrifugation detection. Early in the acquisition process, some samples were also frozen to keep them fresh for several days; we later discovered that frozen milk has a different morphology to cooled milk, which was discernible to such an extent that models could be trained to detect milk that had been frozen with an accuracy of 98%. Indeed, depending on the freezing and thawing rates, there are different processes that may denature MFG: the first is the difference between the crystallization temperatures of water and fat, which causes the growth of ice microcrystals that pierce the MFGM before the fat solidifies. The other is the dehydration of the membranes as the extracellular ice consumes not only ambient water but also bound water [17,18]. Rapid freezing forms smaller crystals that are less damaging; slow freezing, on the other hand, dehydrates and deforms the globules beyond recovery [19].

Prior to imaging, all samples were warmed to 35 °C in order to simulate farm conditions. Next, each sample was divided into two parts: one part was held back as the control, the other went through centrifugation. Both samples were stained using a Sudan III ethanol solution and imaged on several different microscopes (3 to 5): a NIKON Eclipse E-200 (Nikon Corp., Tokyo, Japan) with a Leica Flexacam C1 camera (Leica Microsystems GmbH, Wetzlar, Germany), an Olympus BX53 (Olympus Corporation, Tokyo, Japan) with a Leica Flexacam C1 camera (Leica Microsystems GmbH, Wetzlar, Germany), and an Olympus BX53 with an Olympus DP28 camera (Olympus Corporation, Tokyo, Japan). The observations for all devices were taken within the light field under the brightest light available. Optical magnification was set to 40×. At least five images were taken from each plate with each microscope. Following acquisition, n=42 images were randomly selected for inspection by independent experts, while three were rejected due to insufficient quality (images defocused or obscured). The *χ*^2^ test with a significance level of α=0.05 gave *p*-value of 0.52>α, meaning that 95% of the 25 K collected images were likely to be of good quality. These were the raw inputs to subsequent model training and evaluation procedures.

### 4.2. Augmentation and Normalization

All images captured by the various microscopes were rescaled to a common pixel (px) size of 0.27 µm, then randomly transformed by reflecting natural symmetries of the environment. Crops with an input size of 608 × 608 px were randomly picked out, and their colors were varied using standard color jitter provided by a TorchVision library (brightness, saturation, hue, contrast). We observed that converting the images to gray-scale gave slightly better results, so saturation was set to zero. Since we loaded pre-trained weights for initialization, the architectures were not reduced to single channel, but instead their three channels were fed the same value as the input. There were three non-standard augmentations associated with the microscope setup: (i) Planckian jitter [20], which simulated the variability of the light source; (ii) the simulated chromatic aberration of the optics. The blue and red channels were randomly rescaled in opposite directions around the image center (focal) point (the real aberration evident in one of the microscopes, which had an irregular direction and strength, was somewhat more complicated); and (iii) simulated dust specks accumulating on the lenses. These were large randomly composed gray blobs heavily diffused using Gaussian blur and overlaid on top of the micrograph. The effect was similar to the real dust observed on some of samples. Apart from data augmentation, color normalization was applied to compensate for the different light sources and camera sensors. Each was characterized by a mean RGB and per-channel standard deviation, which were used to center and scale in the color space. These normalization parameters were computed separately for each microscope–camera system, ensuring proper correction of device-specific illumination and sensor characteristics.

### 4.3. Splits and Information Leakages

We used a standard split of 70:15:15 for training, validation, test, respectively. Unless stated otherwise, the splits were generated anew for each training session. Samples were not allowed to cross the splits’ boundaries in order to prevent information leakage. During the training, samples were randomly drawn (with replacement) from the training set so that the ratio between presented categories was equal.

### 4.4. Balancing and Filtering

Unbalanced samples, i.e., those missing either centrifugation or unprocessed parts, were rejected by a principle, because models could have memorized some of their characteristic features and discovered that they always belonged to one category only. For binary model training we also filtered out samples that underwent freezing.

### 4.5. Objectives

The primary modeling objective was to detect centrifugation in milk micrographs. However, we also experimented with alternatives, such as the previously mentioned freezing of samples (which, for the diary, is undesirable due to the milk’s altered structure) and mastitis (which, in turn, is the sole motivation for centrifugation). Training was carried out either for single or multiple objectives. In the latter case, we used power-set labels, which replaces a multi-label classification problem with a multi-class one: that is, a set of *n* independent, binary labels is mapped onto their tensor product, giving an *n*-dimensional hypercube of 2*^n^* corners (power-set labels).

### 4.6. Architectures

Two broad classes of artificial neural network (ANN) architectures were used for modeling: convolutional and attentive. Generally speaking, convolutions are the primary, bottom-up mechanism for pattern recognition, while attention looks for characteristic features top-down. Attentive models are currently ranked at the top for image classification tasks. From both classes we selected some of the best-performing architectures that were small enough to fit into a single 11 GB RTX 2080 Ti GPU. For convolutional ones, we used ResNet-18, ResNet-50 [21], NasNet Mobile [22], Inception v3 [23], and Xception [24]; while for hybrid of convolutional-attentive ones, MaxViT [25] and CoAtNet [26] were used. The memory limitation was particularly difficult to meet for the attentive class, which is memory demanding, therefore our results may not be representative.

### 4.7. Quality Measure

Let the output probability distribution of a classification model of *K* categories be $p_{k}\in \left[0,1\right]$, $k=0\ldots K-1,\;\sum_{k}p_{k}=1$. Further, let $q_{k}\in \left\{0,1\right\},\Sigma_{k}q_{k}=1,$ denote the associated decision indicator vectors. Let ν=0…N−1 run over *N* test cases, labeled by category indicators $\left\{t_{k}^{\nu }\right\}$. Our primary quality measure is the *harmonic average of recalls* (HAR), defined by:

(1) $$HAR:={\left\langle R_{k}^{-1}\right\rangle }_{k}^{-1}.$$

Here, $\left\{R_{k}\right\}$ are per-category recalls, $R_{k}:=\sum_{\nu }^{N}t_{k}^{\nu }q_{k}^{\nu }/N_{k}$. (In binary case $R_{0}=TNR=$ specificity, $R_{1}=TPR=$ sensitivity). Unlike popular measures like *accuracy* and *F*_1_, HAR is invariant to data-bias, and negatively sensitive to model bias in the sense that any imbalance between the recalls $\left\{R_{k}\right\}$ causes HAR to decrease. This is because HAR is maximized by all recalls being equal $\forall_{k,l}R_{k}=R_{l}$, where the value of harmonic average reaches arithmetic one, also known as *balanced accuracy* $BA:={\left\langle R_{k}\right\rangle }_{k}$.

### 4.8. Multi-Image Strategies

Let $\left\{p_{k}^{\nu }\right\}$ be the membership probabilities of category *k* returned by the models. For a subset *S* of all test images belonging to a single sample, define unnormalized products using $P_{k}^{S}:=\prod_{\nu \in S}p_{k}^{\nu }$. Since all other combinations of categories vanish, the normalized probabilities are $p_{k}:=Z^{-1}P_{k}^{S}$, where $Z:=\sum_{k}P_{k}^{S}$. If the model output probabilities are sigmoid functions of logits $p_{k}^{\nu }=\sigma \left(y_{k}^{\nu }\right)$, then the combined probability is the sigmoid of the sum of partial logits:

(2) $$p_{k}=\sigma \left(\Sigma_{\nu }y_{k}^{\nu }\right).$$

This probability is dominated by the most decisive partial outcomes $\left|y_{k}^{\nu }\right|\to \infty$, while indecisive outcomes, $|y_{k}^{\nu }|\to 0$, contribute negligibly. Since at least five images are available for all samples, we hereafter computed HAR using five images, and denote it using “HAR_5_”.

### 4.9. Loss Function and Hyper-Parameters

**Figure 5:**
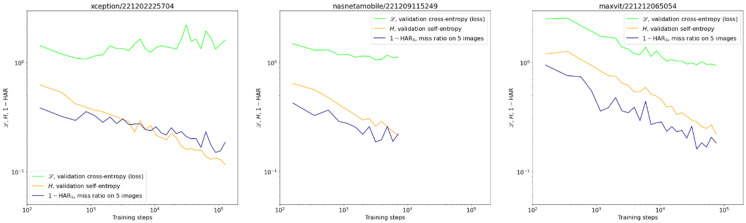
Exemplary validation curves of centrifugation detection for different architectures: cross-entropy *L* (green), self-entropy *H* (orange), and miss rate 1—HAR_5_ (navy). We present the miss rate rather than HAR to make it comparable with the other two quantities on the log-log scale. Notice the upward trend of cross-entropy on the first graph in contrast to the continuing improvement of the miss rate. This situation was no exception.

In order to utilize unlabeled data that were acquired from partner producers, the following semi-supervised loss function was adopted:

(3) $$L:=\sum_{\nu }\left[G{\left(t\right)}^{\nu }H{\left(t,p\right)}^{\nu }+\left(1-G{\left(t\right)}^{\nu }\right)H{\left(p\right)}^{\nu }\right],$$

where $G{\left(t\right)}^{\nu }:=\left(1-K\sum_{k}{\left(t_{k}^{\nu }\right)}^{2}\right){\left(1-K\right)}^{-1}$ is Gini purity; $H{\left(t,p\right)}^{\nu }:=-\sum_{k}t_{k}^{\nu }\log p_{k}^{\nu }$ is cross-entropy, with respect to the targets; and $H{\left(p\right)}^{\nu }:=-\sum_{k}p_{k}^{\nu }\log p_{k}^{\nu }$ is auto-entropy. The cross-entropy term is active for strict targets, while auto-entropy is active for maximally uncertain ones. Training was conducted in random cross-validation *sessions* comprising six models of the same architecture and hyperparameters each, but with different random data splits. For the stopping condition, the minimum of HAR_5_ on validation set is used, with a patience of four. Epoch size is inflated by $2^{1/4}\approx 1.189$ for every pass, i.e., doubling every four passes. The AdaM optimizer [27] with a learning rate of $10^{-4}$ was used. The effective, accumulated batch size was always 64 for a single model update. Input size was 608 × 608 px (164 × 164 µm); three RGB (Figure 5) channels converted to grayscale. 

### 4.10. Class Activation Maps

The class activation maps (CAM) [28] were built to delineate the subset of an image where one of the categories prevail. Micrographs were covered uniformly with overlapping crops of input size 608 × 608 px and a step of 32 px. For each crop model response was obtained, which in a binary case is the difference $y:=y_{p}-y_{n}$ between positive (centrifugation) and negative (unprocessed) logits. This was then extended onto the entire image using weighted averaging using bell-shaped, crop-centered weights. The resolution of this method, understood as the minimal distance between positive and negative extrema, was limited by the input size (608 px).

### 4.11. Class Sensitivity Maps

The most basic sensitivity attribution method is the simple gradient $\nabla_{x}y$ with respect to intensities x of all input pixels regarded as independent dimensions. Unlike the original saliency [29], which used an absolute value, we retained the sign to distinguish between positive and negative contributions. More sophisticated class sensitivity map (CSM) methods provided by the Captum library [30] like Occlusion [31], Deep LIFT [32], Integrated Gradients [33], and Gradient SHAP [34], were abandoned because they either did not yield consistent results, or contradicted the training objective. As in the CAM case, the CSM computed for input size crops were extended to entire micrograph images through weighted averaging on overlapping regions.

### 4.12. Point Spread Functions

Using a sliding 31 × 31 px window, we computed the weighted averages of a 15 px vicinity of each input pixel x and corresponding gradient $\nabla_{x}y$, except image margins. The averages were split into two bins, depending on the central pixel being brighter or darker than its vicinity. The weights were designed to select for a strong peak in the center with a flat vicinity, which would smoothly vanish at the boundaries between the bins. Specifically, we set $w:={\left[\left(x-{\langle x\rangle }_{v}\right)\left(\nabla_{x}y\right)\right]}^{2}/(1+5{\left({\langle x\rangle }_{g}-{\langle x\rangle }_{v}\right)}^{2})$, where ${\langle x\rangle }_{g}$ is the mean luminosity over the whole image, while ${\langle x\rangle }_{v}$ is the mean within pixel vicinity. The total number of accumulated windows was approximately 47 million.

### 4.13. Power Spectral Densities

For each of the top models, power spectral densities (PSD) were calculated by taking a 2D Fourier transformation of the CSM gradient from the 608 × 608 px input window. The spectra were first squared, then averaged over a test set of micrographs in order to enhance common frequencies. The PSD were further reduced into RAPSD 1D plots using radial averaging. Because frequency is inversely proportional to feature size, these plots can be used to decipher which sizes are most relevant for the model when deciding input class.

### 4.14. Classification Based on MFG Distributions

The micrograph images were segmented to extract the diameter, area, circularity, and eccentricity of the MFG. All quantities were binned in a logarithmic scale spanning two orders of magnitude, between 0.1 and 10 µm in diameter. Three methods of image classification were based on this MFG data, the first two using size distributions (MFGD) only: First, we performed a spectral clustering of MFG distributions (MFGD) on the test sets by assuming two clusters for each microscope separately, and using label assignment based on QR factorization [35]. The associations between clusters and classes was based on the mean MFG diameter within a cluster. Second, for hypothesis H3 we computed the ratio $r:=n_{small}/n_{large}$ of small to large MFG counts on sample images. The boundary between categories was set to 2 µm based on consistent cross-points between curves on Figure 3 (left). By assuming a normal distribution of the fluctuations ∆r, the overlap between unprocessed and centrifuged populations for each microscope can be estimated as $FPR=FNR=\frac{1}{2}erfc\left|\left(\mu_{p}-\mu_{n})/(\sigma_{p}+\sigma_{n}\right)\right|$, where $\mu_{c}:=\langle r_{c}\rangle$, $\sigma_{c}^{2}:=\langle {∆r}_{c}^{2}\rangle$, are the means and variances for the small/large ratios for category *c* of the negative (c=n) and positive (c=p) samples. Since FPR and FNR are equal, so are the estimated accuracy and HAR measures:

(4) $$HAR=A=\frac{1}{2}+\frac{1}{2}erf\left|\frac{\mu_{p}-\mu_{n}}{\sigma_{p}+\sigma_{n}}\right|$$

Third, a logistic classifier was trained given statistics of MFG data (diameter, area, circularity and eccentricity) within an image.

## 5. Conclusions

This study demonstrates that deep learning models can detect centrifugation-related alterations in raw milk that remain below the threshold of human visual perception. While our results indicate that changes associated with MFGM and solid non-fat components contribute substantially to the discriminative optical signatures, these findings should be interpreted with caution. The evidence for micellar reorganization is based on indirect optical cues rather than direct physicochemical measurements and therefore represents an inferred, not experimentally confirmed, mechanism. The integration of bright-field microscopy with AI-based analysis provides a promising framework for developing objective, image-driven diagnostics of centrifugation-induced adulteration. Future research should include multi-laboratory validation to assess robustness across different optical systems, sample origins, and environmental conditions. Scaling the methodology toward industrial implementation—for example, through automated screening modules embedded in routine quality-control pipelines—may further enhance its practical relevance. Finally, to strengthen the mechanistic interpretation of the observed optical patterns, complementary physicochemical biomarkers (such as rheological, spectroscopic, or structural assays, including TEM or DLS) will be essential. Such multimodal validation would help determine the extent to which computational predictions correspond to underlying molecular transformations, ultimately improving the reliability and interpretability of AI-based milk authentication tools.

## Figures and Tables

**Table 1:** Complete confusion matrix from the Experts Trial. HU = healthy-unprocessed, HC = healthy-centrifuged, MU = mastitis-unprocessed, MC = mastitis-centrifuged.

		Prediction			
		HU	HC	MU	MC
GT	HU	5	10	6	9
	HC	10	13	3	5
	MU	12	6	6	9
	MC	14	16	0	4

**Table 2:** Comparison of different architectures in the simultaneous detection of centrifugation and freezing. The values are HAR_5_ measured on the test sets in random, cross-validation sessions of six models. Uncertainties are standard deviations within the session.

Architecture	Feature	Best in Session (%)	Average (%)
*CoAtNet*	centrifugation	92	77 ± 9
	freezing	91	76 ± 10
*Inception-v3*	centrifugation	89	82 ± 6
	freezing	89	81 ± 7
*MaxViT*	centrifugation	85	73 ± 14
	freezing	85	71 ± 16
*NasNet Mobile*	centrifugation	86	80 ± 4
	freezing	86	79 ± 5
*ResNet-18*	centrifugation	90	78 ± 6
	freezing	88	76 ± 7
*ResNet-50*	centrifugation	87	80 ± 4
	freezing	86	79 ± 3
*Xception*	centrifugation	97	81 ± 8
	freezing	97	81 ± 8

## Data Availability

The original contributions presented in this study are included in the article. Further inquiries can be directed to the corresponding author.

## Abbreviations

The following abbreviations are used in this manuscript:

ML	machine learning
AI	artificial intelligence
MFG	milk fat globule
MFGM	milk fat globules membrane
NIR	near-infrared
ANN	artificial neural network
HAR	harmonic average of recall
CAM	class activation map
CSM	class sensitivity map
PSD	power spectral density
MFGD	milk fat globule distribution
RAPSD	radially averaged power spectral density
PSF	point-spread function
